# Effects of Consumer Interactions on Benthic Resources and Ecosystem Processes in a Neotropical Stream

**DOI:** 10.1371/journal.pone.0045230

**Published:** 2012-09-28

**Authors:** Michael C. Marshall, Andrew J. Binderup, Eugenia Zandonà, Sandra Goutte, Ronald D. Bassar, Rana W. El-Sabaawi, Steven A. Thomas, Alexander S. Flecker, Susan S. Kilham, David N. Reznick, Cathy M. Pringle

**Affiliations:** 1 Odum School of Ecology, University of Georgia, Athens, Georgia, United States of America; 2 Department of Biology, Drexel University, Philadelphia, Pennsylvania, United States of America; 3 École Normale Supérieure of Paris, Paris, France; 4 Department of Biology, University of California Riverside, Riverside, California, United States of America; 5 Ecology and Evolutionary Biology, Cornell University, Ithaca, New York, United States of America; 6 School of Natural Resources, University of Nebraska, Lincoln, Nebraska, United States of America; University of Southampton, United Kingdom

## Abstract

The effect of consumers on their resources has been demonstrated in many systems but is often confounded by trophic interactions with other consumers. Consumers may also have behavioral and life history adaptations to each other and to co-occurring predators that may additionally modulate their particular roles in ecosystems. We experimentally excluded large consumers from tile periphyton, leaves and natural benthic substrata using submerged electrified frames in three stream reaches with overlapping consumer assemblages in Trinidad, West Indies. Concurrently, we assessed visits to (non-electrified) control frames by the three most common large consumers–primarily insectivorous killifish (*Rivulus hartii*), omnivorous guppies (*Poecilia reticulata*) and omnivorous crabs (*Eudaniela garmani*). Consumers caused the greatest decrease in final chlorophyll *a* biomass and accrual rates the most in the downstream reach containing all three focal consumers in the presence of fish predators. Consumers also caused the greatest increase in leaf decay rates in the upstream reach containing only killifish and crabs. In the downstream reach where guppies co-occur with predators, we found significantly lower benthic invertebrate biomass in control relative to exclosure treatments than the midstream reach where guppies occur in the absence of predators. These data suggest that differences in guppy foraging, potentially driven by differences in their life history phenotype, may affect ecosystem structure and processes as much as their presence or absence and that interactions among consumers may further mediate their effects in these stream ecosystems.

## Introduction

Top-down control of food resources by consumers has been a central tenet in ecology for the past five decades [1]–[3] and continues to stimulate research today [4]. Such research has contributed to our understanding of ecological interactions in applications as diverse as wildlife re-introductions [5] and fishery declines [6] and in a variety of environments including marine [7] and terrestrial [8] systems. In rivers, trophic roles of consumers are often predicted to vary along the longitudinal continuum as energy supply shifts from allochthonous litter in forested headwaters to autochthonous production in more open systems [9] and as consumer diversity increases, the potential for complex trophic interactions among consumers may also increase [10]. In addition, some of the resulting trophic interactions may affect ecosystem processes (i.e., rates of change of chemical or biotic variables) without observable changes in ecosystem structure (i.e., abundance, concentration or biomass of chemical or biotic variables) [11], underscoring the need for examining both structural and process responses for identifying the changing roles of consumers among different assemblages.

While numerous studies have revealed the role of consumers in a specific context, fewer studies have accounted for the phenotypic (e.g., trophic, behavioral, etc.) variation in the same consumer in response to interactions with other consumers. For example, organic matter resource responses to consumers are often confounded by multiple top-down effects [12] or consumer interactions with bottom-up effects [13]. Some studies address these confounding factors by isolating target consumers in enclosures in the absence of other consumers [14]. By doing so, researchers can address the specific impacts of one consumer, but the potential consequences of interactions with other consumers may be overlooked. To evaluate potentially modulating ecological roles of consumers in ecosystems, investigators could take advantage of naturally overlapping assemblages exhibiting multiple ecological forces, such as interspecific competition and predation, that structure their interactions [15].

In Trinidadian streams, naturally overlapping consumer assemblages are separated by barrier waterfalls, where upstream reaches are dominated by killifish (*Rivulus hartii* Boulenger) and crabs (*Eudaniela garmani* Rodriguez and Diaz), midstream reaches by killifish, crabs and guppies (*Poecilia reticulata* Peters), and downstream reaches have the aforementioned taxa in addition to piscivorous fishes [16], [17]. Aquatic consumers in Trinidad frequently serve as model systems for studies of evolutionary dynamics [18], social behavior [19] and community interactions [20]. Because of these broad ecological applications and the potential for similar assemblage combinations to reoccur throughout the Neotropics [21]–[23], this system provides an excellent template on which to examine top-down effects that could be tested in a variety of other streams and consumer-driven systems in general.

In addition to a gradient of increasing assemblage complexity in the Trinidadian system described above, there is also known local adaptation of some of the key consumers (Table 1). For example, killifish that occur as the only fish in headwater streams exhibit higher population densities, reproduce later in life and have lower reproductive allotment than in streams where they co-occur with guppies [24] and even greater differences in life history adaptations between isolated headwater populations and where they also occur with fish predators [25]. Also, guppies from midstream reaches without predators generally occur at higher population densities, produce fewer, larger offspring [26] and feed mostly on algae and detritus [27] compared to guppies co-occurring with predators in downstream reaches which tend to have lower densities, produce more, smaller offspring [26] and forage more on benthic invertebrates [27]. Recent evidence from mesocosm experiments suggest that these differences in diet, life history traits and density can significantly affect resource standing biomasses and processes [28], but it remains unclear whether these intraspecific differences are important for ecosystem structure and processes in nature. It has recently been argued that top-down effects can also interact with local adaptation to predators. For example, landlocked alewives altered the structure of zooplankton assemblages by removing all large-bodied species, then evolved more closely-spaced gill-rakers that enabled them to better exploit the now predator-adapted zooplankton assemblage [29]. These interactions between ecological and evolutionary processes hypothesized in lakes [29] and demonstrated in mesocosm experiments [28] may be widespread. If so, factors like local adaptation to interspecific interactions that include local differences in their impacts on the ecosystem must also be incorporated into our evaluation of top-down effects in natural assemblages.

**Table 1:** Ecological and life history differences of 2 focal consumers in 3 reaches.

Species	Trait/Behavior/Interaction	UPSTREAM	MIDSTREAM	DOWNSTREAM	Reference
Guppy	Diet	NA	35% Inverts, 49%Detritus, 5% Algae	65% Inverts, 32% Detritus, 1% Algae	[27]
	Maturity/size	NA	Late/large	Early/small	[32]
	Reproductive Effort	NA	Fewer, larger offspring	More, smaller offspring	[54]
	Predators	NA	Killifish	Wolf fish, sardines, coscarobs,killfish	[16]
	Competitors	NA	Killifish	Killfish, tetas, catfish	[16]
	Microhabitat/activity	NA	All/day	Shallow pools/day, Shallowedges/night	[78]
	Density	NA	High	Low	[26]
Killifish	Diet	Dipteran larvae & adults, ants	Dipteran larvae & adults, ants	Dipteran larvae & adults, ants	[58]
	Maturity/size	Late/large	Intermediate	Early/small	[25]
	Reproductive Effort	Fewer eggs	Intermediate	More eggs	[25]
	Predators	NA	NA	Wolf fish	[16]
	Competitors	NA	Guppies	Sardines, coscarobs, guppies	[16]
	Microhabitat/activity	Ubiquitous/24 hours	Deep pools/day, Shallow edges/night	Isolated stream margins/nocturnal	[58]
	Density	High	Intermediate	Low	[24]

Here we examine how top-down effects of ecosystem structure and processes vary among three different consumer assemblages in a Trinidadian stream system. We used an experimental approach to compare the direct top-down effects of consumers on benthic ecosystem structure (periphyton biomass and invertebrate biomass and assemblage composition) and processes (rates of leaf decay and periphyton accrual) of each reach. Because of their ecological and evolutionary importance in this system, we used electrified frames to selectively exclude all large consumers (particularly guppies and killifish) from our experimental plots in stream pools in order to quantify their impact on lower trophic levels and pool-scale ecosystem processes. We predicted that periphyton accrual and biomass would be significantly reduced in midstream reaches where guppies and other large consumers occur without fish predators (i.e. downstream). Likewise, we predicted that leaf decay rates would be fastest in the upstream reach where large shredders are abundant and competitive and predatory interactions are minimal. We predicted the greatest effect of consumer assemblages on primary productivity and benthic invertebrate responses in the downstream reach because greater trophic diversity, particularly omnivores, should exert the greatest top-down effects (negative) on primary producers and primary consumers. In addition, we examined two benthic invertebrates specifically known to be important components of guppy diets (Ephemeroptera and Diptera) and predicted the largest consumer effects on those taxa in systems with predation-adapted guppies (downstream). Because consumer density can also contribute to variation in resource levels [28], we quantified consumer visitation to control frames as a proxy for local densities.

## Methods

### Ethics Statement

Animal handling for this study was approved by the University of Georgia’s Institutional Animal Care and Use Committee Protocol (A2007-10107-0, Catherine Pringle and Michael Marshall PIs). This study was performed on non-protected state (downstream) and private (upstream and midstream) lands in Trinidad. Permission to work on the private land was granted by the landowner, Euston Devonish, of Toco, Trinidad. No specific permits were required for the described field study at these locations or for these activities. This study did not involve endangered or protected species.

### Site Description

We conducted our experiments in streams within the Guanapo River watershed in the montane Northern Range in Trinidad, West Indies. Based on previous biotic surveys [16], [17], [30], we selected stream reaches characterized by three distinct overlapping consumer assemblages: (1) an upstream reach containing the killifish *Rivulus hartii*, and the crab *Eudaniela garmani*; (2) a midstream reach dominated by the aforementioned taxa, the abundant guppy *Poecilia reticulata*, and the Pimelodid catfish *Rhamdia quelen* (Quoy and Gaimard) in very low numbers; and (3) a downstream reach also with five larger fish species including guppy predators such as the common wolf fish *Hoplias malabaricus* (see Table S1 for a complete list). The wolf fish and other predaceous fish have been shown to be key drivers of population demographics, foraging behavior and the evolution of life history traits in guppies [27], [31], [32] and killifish [25].

Each reach was isolated from the other reaches by intervening barriers and waterfalls that have likely maintained local assemblages and associated trophic interactions (Table 1) for at least two decades [16]. We selected adjacent upstream and midstream reaches within 200 m of each other and the nearest accessible piscivore-containing reach ∼3 km downstream to minimize abiotic differences between reaches and manage logistical challenges of access to sites. Sites had similar physicochemical characteristics (Table S2), although the midstream reach was significantly shadier than the other reaches and the downstream reach had warmer water temperatures, higher dissolved oxygen concentrations, slower water velocities and higher discharge than the other two reaches. Nutrient concentrations were also slightly higher in the downstream reach, but due to low light conditions in all reaches, were not expected to significantly affect primary productivity metrics [33].

### Electric Exclosures and Experimental Design

We excluded large consumers using electrified wire frames [34]. Exclosures were constructed of two concentric rectangles of 8-gauge (3.26 mm diameter) solid copper wire, connected by plastic cable ties (outer rectangle 25 cm × 50 cm, inner 8 cm × 30 cm). Paired control (non-electrified) and electric frames were installed in pools at equal depths and within a standardized range of flow velocities between 0.01 and 0.09 m·s^−1^ (Table 1). We used Speedrite “Viper” 5000 fence chargers (Tru-Test Limited, Auckland, New Zealand) on low power and slow pulse settings resulting in energy output of about 3 joules at ∼2-second intervals. Twelve-volt, 33-Amp hour, batteries provided continuous power to the fence chargers during the three or four week treatment period. The effectiveness of electrification in water is a function of fence charger power and animal size [35]. In the context of our experimental manipulation, “large consumers” refer to animals big enough to be directly affected (i.e. excluded) by the electric treatments. We selected the fence charger and power setting to preferentially target all of the fishes in each study reach. Because of the size of some crabs in our sites, large crabs were also likely affected by the electrification. We confirmed this effectiveness of exclosures in the field by observing invertebrates and fish in and around the electrified frame.

We ran experiments in five replicate pools (one pair of frames per pool) in each reach (150–200 m in length). Due to the logistical constraints of a limited number of fence chargers and the difficulties of managing exclosures simultaneously at more than 2 sites in a rugged landscape, we ran experiments concurrently in the upstream and midstream reaches between mid-February to mid-March 2008 and immediately following in the downstream reach in April 2008 (see specific dates in Table S2). This period is during the dry season when hydrological conditions are relatively stable and physical disturbance was minimal. We intended to run the experiments for a full 4-week period in all reaches, but had to take final samples and retrieve equipment at the end of the third week in the downstream reach due to vandalism.

### Leaf Decomposition

We used bagless packs of fast-decomposing fresh leaves to assess consumer effects on decomposition. Fresh-picked black stick (*Pachystachys coccinea* Nees) leaves were dried at 40°C for at least 3 days. Fresh leaves are generally considered to be a high quality resource in aquatic food webs [36]. They represent natural input from storms and natural treefalls [37] and have been used here to facilitate short-term estimates of decomposition. Leaves were grouped in batches of 3–4 g, weighed to the nearest 0.01 g and clipped together at the petiole using a binderclip [38] to allow access by larger shredding consumers common in Trinidadian streams (e.g. crabs). We initially placed 10 leaf packs on day 0 in both control and electrified frames. For logistical reasons we sampled 2 leaf packs from each frame on days 3 and 7 in upstream and midstream reaches and days 2 and 6 in the downstream reach and weekly thereafter in all 3 reaches. Due to the highly labile nature of black stick leaves, most of the material degraded in control treatments by the second week, thus we used data from the first 2 weeks to calculate leaf decay rates for each frame. We added more leaf packs to frames after the second week to maintain similar habitat conditions throughout the experimental period, but only summarize decomposition of the original set of leaf packs here. Retrieved leaf packs were rinsed over a 250-µm sieve and all recognizable leaf particles were placed in pre-weighed paper bags. Bags were dried at 40°C for at least 2 days and weighed to the nearest 0.01 g. We calculated the percent remaining leaf dry masses using initial and final measurements and natural log-transformed the data for statistical analysis.

### Periphyton Biomass and Accrual

We estimated consumer effects on periphyton by measuring chlorophyll *a* and ash-free dry mass (AFDM) through time on unglazed ceramic tiles incubated in experimental frames. Tiles were ashed at 500°C for at least 2 hrs prior to deployment to eliminate any organic matter from previous use. Because tiles were ashed prior to deployment, we assumed initial chlorophyll *a* and AFDM were negligible. We secured 8 tiles to experimental frames using binder clips attached by small cable ties on day 0. Two tiles were retrieved from each frame on days 7, 14, 21 and 28 in upstream and midstream reaches and days 6, 13 and 20 in the downstream reach where vandalism necessitated the last sampling date to be on day 20. Retrieved tiles were scraped with a steel wire brush and the resulting slurry was homogenized and subsampled for chlorophyll *a* and AFDM [39]. Chlorophyll *a* subsamples were pipetted onto a 25-mm diameter glass fiber filter (1.0 µm) and AFDM subsamples onto a pre-ashed, pre-weighed 47-mm diameter glass fiber filter (0.7 µm). Chlorophyll filters were frozen for at least 24 hours to facilitate cell lysing then extracted using 90% ethanol incubated at room temperature for 24 hours. We measured fluorescence using a Turner Aquafluor handheld fluorometer (Turner Designs, Inc., Sunnyvale, CA, USA) fitted with a chlorophyll-specific wavelength channel. We did not correct for phaeo-pigments because we intended to only make within-study comparisons of relative consumer effects among treatments.

### Benthic Invertebrates

We sampled natural benthic substrate from all frames using a pipe core (91.6 cm^2^). We stirred the contents in the pipe core and used a dip cup to remove water and suspended invertebrates and benthic debris retained on a 63-µm mesh net [40]. We took one pipe core sample in each frame which represented 7.3% of the total frame area or ∼22% of natural benthic area unaffected by the other sample substrates (tiles and leaves) within the frames. Benthic invertebrates were collected on day 28 in the upstream and midstream reaches and on day 21 in the downstream reach (see above explanation). We estimated individual invertebrate biomasses of animals retained on a 250-µm sieve using length-mass regressions for insects [41, T. Heatherly, *personal communication*] or volume-mass formulas for non-insect invertebrates [42]. We also separately analyzed results for Ephemeroptera and Diptera, two insect orders particularly likely to be impacted by consumer foraging [27], [43]. Although other studies have successfully used the same fence charger model on the higher power (5 J) setting to exclude small invertebrates [44], behavioral observations and comparisons between control and treatment benthic samples suggest that electrification had minimal direct effects on small invertebrates in our study.

### Observations of Consumer Visitation

We quantified diurnal visitation by consumers to control treatments over two periods during the first week and one period each week thereafter. We made nocturnal observations for two periods (during weeks 1 and 2) in the upstream and midstream reaches and during one period (during week 2) in the downstream reach. At each pool, an observer positioned ∼1 m from the frame waited quietly for 5 min after arriving at the site, which is long enough for consumers to resume normal activities [26]. After 5 min, we recorded species identification, number of individuals and size class once per minute for 10 min for a total of 15 hrs of observation across all sites. We calculated visitation rate by dividing number of individuals by the control frame area and number of min observed and converted it to an hourly rate resulting in units of number of individuals m^−2^·hr^−1^. Fish did not enter the exclosures or immediately left the electrified area upon shock. Small crabs (<2.5 cm) were occasionally found dead in exclosures and immediately removed to prevent localized nutrient enrichment to leaves and tiles due to their decomposition.

### Statistical Analysis

We analyzed the effect of excluded consumers on the structural and process variables using a split-plot design (Methods S1) and planned comparisons. We used planned comparisons to calculate and test differences in consumer effects within and between reaches. Consumer impact (CI) indices have previously been calculated as -ln(non-electrified exclosure/electrified exclosure) [45] which give a dimensionless index that can be compared across response variables of different units. We use the reverse ratio, ln(non-electrified exclosure/electrified exclosure), so that positive CI values indicate increase in response variable with consumers, whereas negative CI values indicate a decrease. In the context of planned contrasts, these consumer indices can be calculated as the difference between the split-plot and split-plot by whole-plot interaction effects (partial interaction contrasts) of the linear model when the dependent variable has been natural log-transformed. To calculate within reach contrasts, we coded the contrast matrix as −1 and 1 for the control and electrified exclosures (split-plot effect) and the corresponding interaction effects for the level of the reach (whole plot) as −1 and 1 with other reaches as zeros. To calculate differences in the consumer indices between reaches, we subtracted the within reach contrast matrix from the other for whichever between reach test we were interested in and designated this variable as ΔCI. All analyses were conducted using the linear mixed model procedure in SAS [46].

## Results

### Consumer Visitation to Control Frames

The three study reaches displayed clear differences in consumer assemblages (Table S1). We observed killifish and crabs in the upstream reach, killifish, crabs and guppies in the midstream reach, and crabs and guppies in the downstream reach (Table 2). We also observed a single catfish (*Rhamdia*) visiting a control frame twice during one observation period in the midstream reach. Although clearly present in stream margins and under natural leaf packs, killifish were never observed in the control frames in the downstream reach during our visitation estimates. We observed other fish species in stream margins under hanging vegetation and boulders during the experiment in the downstream reach, including the ambush fish predator, *Hoplias*, and the two armored catfish periphyton grazers, *Hypostomus* and *Ancistrus*, but none of these other taxa entered the control frames during our observations.

**Table 2:** Mean (±1SE) visitation by guppies, killifish and crabs in all three stream reaches during day and night.

		Visitation rate (individuals m^−2^·hr^−1^)		
Period	Visitor	Upstream	Midstream	Downstream
Day	Guppy	NA	29.3(3.5)	25.3(5.3)
	Killifish	12.8(2.9)	1.3(0.5)	0.0(0.0)
	Crab	0.0(0.0)	0.0(0.0)	0.2(0.1)
Night	Guppy	NA	2.2(1.5)	12.4(10.2)
	Killifish	27.0(6.3)	5.2(1.9)	0.0(0.0)
	Crab	24.7(5.4)	4.8(4.8)	12.0(12.0)
Total Daily Mean	Guppy	NA	15.7(3.3)	18.8(4.7)
	Killifish	19.9(2.9)	3.3(0.7)	0.0(0.0)
	Crab	12.1(2.3)	2.4(1.3)	6.1(2.3)

Total daily mean was based on 12 hr diel periods and weighted to compensate for fewer night observations.

NA = not applicable (guppies not present in upstream reach).

### Periphyton Responses

**Figure 1:**
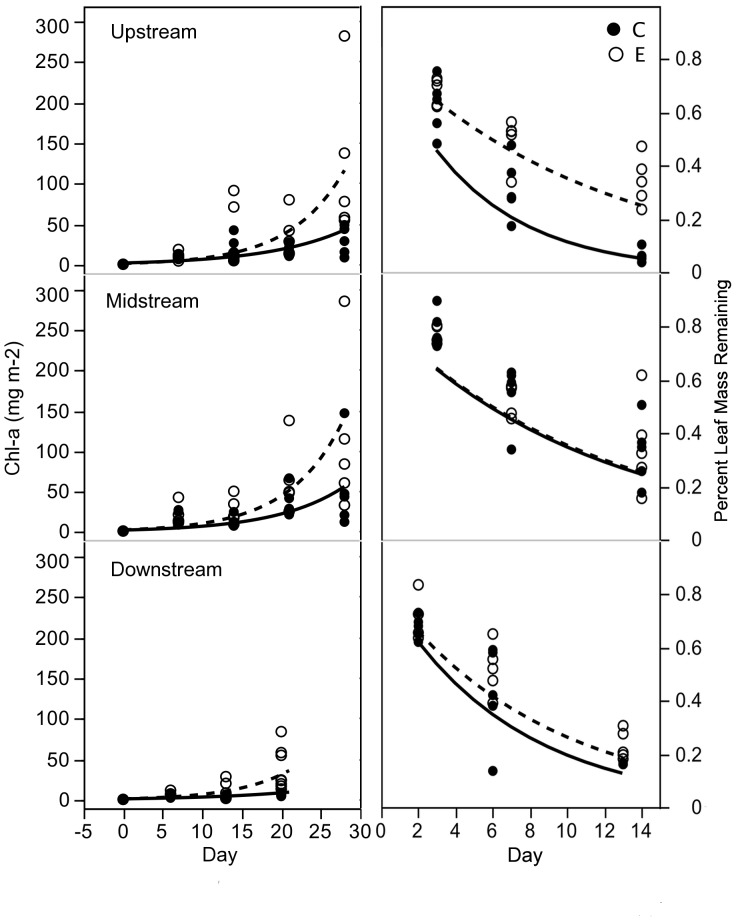
Time series plot for chlorophyll a and leaf matter. Raw data (circles) and predicted values (curves) from mixed model analyses of periphyton chlorophyll *a* accrual rates (left panels) and leaf decomposition (right panels). Responses in controls (C) are solid symbols and lines, exclosures (E) are hollow symbols and dashed lines. Periphyton biomasses were evaluated at day 20 or 21 of the experiment.

**Figure 2:**
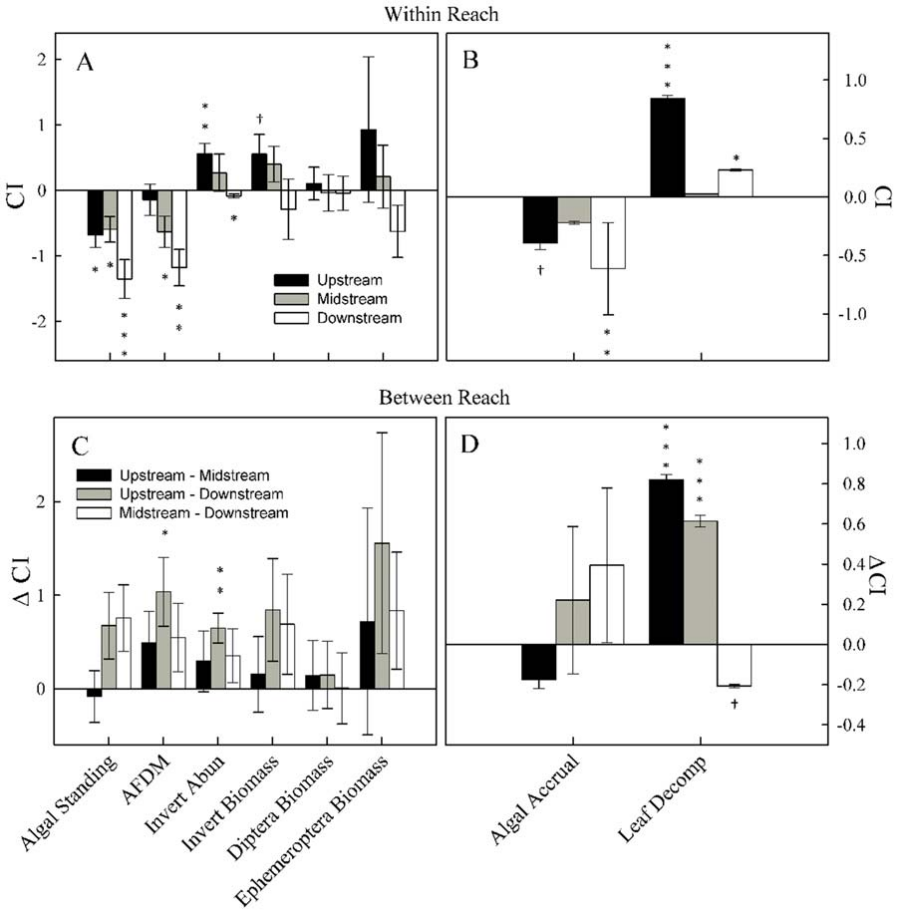
Consumer Impacts (CI) for each reach (Within) and the differences (ΔCI) between reaches (Between). All CI values are calculated from planned comparisons from linear models except slope estimates, which were calculated from the predicted slopes from the fixed and random effects models (see text). Positive values indicate increase in response variable with consumers, whereas negative values indicate a decrease. Each bar is the mean (±1 SE) of 5 replicates. All significance values are from planned contrasts from linear model with ***P<0.001, **P<0.01, *P<0.05, †*P*<0.10.

Consumers significantly decreased final chlorophyll *a* on tiles in the control relative to exclosure treatments within all reaches, while their effects on periphyton accrual rates (Fig. 1, left panels) and periphyton AFDM (Fig. 2A) were more variable. Despite consistent reductions in final chlorophyll *a* biomass in controls relative to exclosures within all reaches (Table 3), chlorophyll *a* accrual rates were significantly reduced by consumers only within the downstream reach (*F*_1,122_ = 7.08, *P*<0.01) and marginally reduced in the upstream reach (*F*_1,122_ = 3.82, *P* = 0.053). Consumers also significantly reduced periphyton AFDM in control relative to exclosure treatments within the midstream (*F*_1,10_ = 6.97, *P*<0.05) and downstream reaches (*F*_1,10_ = 18.10, *P*<0.01). Although chlorophyll *a* biomass in control treatments was highest in the midstream reach (Table 3), consumer impact (CI) indices for periphyton structural (Fig. 2A, chlorophyll *a* and AFDM) and process (Fig. 2B, chlorophyll *a* accrual rate) responses were the most negative (*i.e.*, lower when consumers are present) in the downstream reach where consumer diversity is greatest.

**Table 3:** Mean (±1SE) response values in control (C) and exclusion (E) treatments for all three reaches. Significantly larger values (*P*<.05) for each treatment pair indicated in bold.

		Chl *a* Biomass	Chl *a* Accrual	Periphyton AFDM	Invert Biomass	Invert Abundance	Leaf Decay
Reach	Treatment	(mg chl *a*·m^−2^)	(mg chl *a*·m^−2^·d^−1^)	(g AFDM·m^−2^)	(mg DM·m^−2^)	(# m^−2^ X100)	(d^−1^)
UP	C	20.7(3.2)	1.01(.16)	79.2(20.5)	**932(97)**	29.2(2.4)	**0.211(.016)**
	E	**36.1(12.2)**	1.89(.73)	88.4(18.1)	613(179)	17.1(2.3)	0.073(.011)
MID	C	36.7(8.2)	1.57(.40)	73.9(9.4)	721(78)	28.9(5.4)	0.087(.013)
	E	**65.1(19.2)**	2.83(.93)	**155.9(40.6)**	526(114)	24.9(6.5)	0.082(.019)
DOWN	C	7.0(0.5)	0.25(.01)	16.3(2.5)	493(171)	8.2(1.3)	**0.133(.001)**
	E	**37.3(12.4)**	**1.49(.62)**	**50.2(8.0)**	**599(177)**	8.5(1.7)	0.109(.010)

Chlorophyll *a* biomass and periphyton AFDM (ash-free dry mass) collected in week 3 for all reaches.

Invertebrate biomass and abundance collected after 4 weeks in upstream and midstream reaches, after 3 weeks in downstream reach.

### Leaf Decomposition Responses

**Figure 3:**
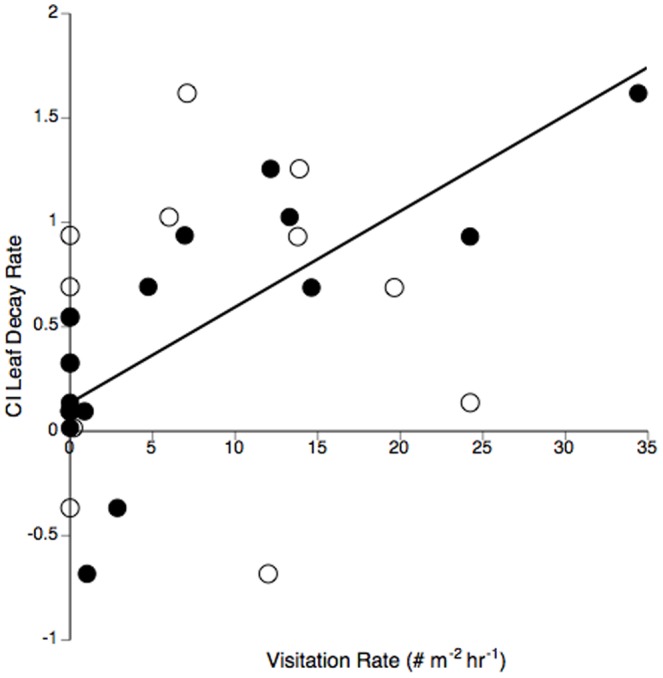
Consumer Impact (CI) of leaf decay rate plotted against visitation rate across all study reaches. Killifish plotted as solid symbols. Crabs plotted as hollow symbols. Line is a linear fit (*P*<.001, r^2^ = .59) for killifish. The relationship for crabs was not significant. Positive CI values indicate increase in response variable with large consumers present, whereas negative values indicate a decrease.

Black stick leaves decomposed rapidly in both exclosures and controls and almost completely disappeared by the third week in all reaches (Fig. 1, right panels) resulting in leaf decomposition rates ranging from 0.027 to 0.26 d^−1^. Within reach leaf decomposition rates were significantly faster by 3-fold in the control than exclosure treatments in the upstream reach (*F*_1,72_ = 117.33, *P*<0.001, Table 3) and by 0.3-fold in the downstream reach (*F*_1,72_ = 6.64, *P*<0.05, Table 3). Within reach decomposition rates were not significantly different between treatments in the midstream reach (Table 3). The CI index of leaf decomposition rates was strongest (most positive) in the upstream reach (Fig. 2B), suggesting consumers from the least and most diverse consumer assemblages facilitated leaf decay. The CI of leaf decomposition was significantly positively related to killifish visitation (Fig. 3, solid symbols), suggesting killifish facilitated leaf decay in control treatments relative to exclosures.

### Benthic Invertebrate Responses

Consumers significantly increased benthic invertebrate abundance by 1.7 X (*F*_1,11_ = 13.13, *P*<0.01) and marginally increased invertebrate biomass (*F*_1,11_ = 3.44, *P* = 0.088) in control treatments relative to exclosures in the upstream reach (Table 3). A 6-fold greater mean ostracod biomass in control (0.399±0.088SE g m^−2^) than exclosure (0.066±0.028SE g m^−2^) treatments accounted for most of this consumer effect. There were no significant effects of consumers on benthic invertebrate abundance or biomass in the midstream reach. Consumers significantly decreased benthic invertebrate abundance (*F*_1,11_ = 7.421, *P*<0.05), but not biomass, in control relative to exclosure treatments in the downstream reach (Table 3), suggesting consumers were directly consuming benthic invertebrates in the reach where the consumer assemblage is most trophically diverse. Consumer impact (CI) indices shifting from most positive (increases in benthic invertebrates with larger consumers) in the upstream reach to the most negative (decrease in invertebrates with larger consumers) in the downstream reach (Fig. 2A) support this trophic diversity mechanism.

Benthic invertebrate assemblages were dominated numerically by collector dipteran larvae (mostly Chironomidae) and collector Ostracods in upstream (64.7% and 26.1%, respectively) and midstream (66.9 and 26.7%, respectively) reaches and by collector-gatherer ephemeropteran nymphs (34.8% Leptohyphidae) and dipteran larvae (17.5% Chironomidae, 9.9% Ceratopogonidae) in the downstream reach. Benthic invertebrate biomasses were dominated by chironomid larvae and Ostracods in upstream (43.5 and 30.1%, respectively) and midstream (26.8 and 53.9%, respectively) reaches and by primarily grazer coleopteran larvae (30.8% Psephenidae, 19.0% Elmidae) in the downstream reach. Mean total biomasses of Diptera were 2- and 3-fold greater in controls in midstream and upstream reaches, respectively, than the downstream reach, but there were no significant exclusion effects on Diptera in any reach (Fig. 2A). Within reach mean total biomass of Ephemeroptera was 2 times higher in exclusion (101 mg DM·m^−2^) than control (47.7 mg DM·m^−2^) treatments in the downstream reach (Fig. 2A), but not significantly different in the other reaches. Some rare benthic invertebrates appeared to be excluded from electrified treatments in some reaches (Table S3). Because all of these exceptions also occurred in only 1 of 5 replicate pairs of frames in only 1 reach, we suggest there were negligible effects of the electrification on the differences in total invertebrate abundances between sites.

## Discussion

We experimentally quantified direct consumer effects on benthic ecosystem structure and processes in three stream reaches with overlapping consumer assemblages in a montane Neotropical watershed. Our results indicate that all 3 focal large consumers (guppies, killifish and crabs) have a strong influence on periphyton AFDM, periphyton chlorophyll *a* biomass, chlorophyll *a* accumulation rates and leaf processing rates, but weaker influence on benthic invertebrate abundance and biomass. Interestingly, the magnitude and direction of those responses depended on which consumers were present and apparent community interactions occurring among them (Table 1). Thus, we discuss each ecosystem response variable in the context of differences in consumer assemblages using our knowledge of their diets and behaviors and well-studied local community interactions from the literature.

### Mechanisms for Periphyton Response

Typically primary resources become more limiting where interactions among consumers are most diverse [47]. For example, foraging by omnivorous fishes significantly reduced algal and fine organic matter standing crops in diverse Neotropical streams [48]–[50]. We also observed the greatest consumer effect on chlorophyll *a* biomass (Fig. 2A) and accrual rate (Fig. 2B) in the downstream reach and periphyton AFDM (Fig. 2A) in the midstream and downstream reaches where consumer interactions are more diverse than our upstream reach. Differences in CI (ΔCI) for AFDM were greatest between upstream and downstream reaches (Fig. 2C), suggesting downstream consumers had much higher demand for periphyton resources than upstream consumers. Chlorophyll *a* biomass was largely associated with light availability in a survey in similar streams [51]. Because paired exclosure and control frames were positioned within 1 m of each other and blocked by pool, it is unlikely that periphyton responses were due to differences in light or other abiotic factors between treatments: in fact, chlorophyll *a* biomass in the absence of large consumers was highest in the shadiest (midstream) reach (Table 3). Phosphorus concentrations were above the theoretical threshold for nutrient limitation of 15 µg P L^−1^ and ammonium concentrations were below the theoretical threshold for N limitation of 50 µg N L^−1^ [52] in all 3 reaches in our study. Because we did not measure nitrate during our experiments, it is not possible to establish that any reach was N limited. However, total dissolved inorganic nitrogen (NO_3_ + NH_4_) did exceed theoretical N limitation in 3 homologous reaches in the same watershed in a related study during another year [33], suggesting that none of our reaches were limited by either N or P. The same study found stronger evidence that due to ubiquitous tropical mountain forest canopy cover, Trinidadian headwater streams are more commonly light than nutrient limited [33]. Despite relatively high consumer diversity in the downstream reach, guppies were the dominant consumers observed in control treatments, and possibly contributed to the periphyton response in midstream and downstream reaches.

One potential mechanism driving the difference in periphyton accrual and biomass between midstream and downstream reaches is intraspecific variability in the diets between the different guppy phenotypes. Guppies commonly forage on benthic periphyton in the wild and their diet mainly consists of unidentifiable detritus [28], but guppies from reaches without predators consume a higher proportion of algae than those from reaches with predators, which tend to feed mostly on invertebrates [27], [28]. Correspondingly, guppies from reaches without predators can dramatically reduce chlorophyll *a* biomass and benthic organic matter, while those from reaches with predators can release algae from benthic invertebrate grazing in mesocosms [28]. Many predation-selected guppy life history traits are heritable [32], have a genetic basis [31] and are inducible by transplanting guppies from high to low predation streams [18] and may apply to guppy diets in our streams. Assuming these diet differences were present in our experimental reaches, we would expect the greatest impact on periphyton in the midstream reach where guppies should be more algivorous (see Table 1). This was not the case for our experiment, suggesting that periphyton response was not driven by differences in guppy phenotype alone.

Differences in periphyton responses between lab experiments and field experiments are not uncommon. For example, in a meta-analysis of 865 experimental studies periphyton biomass is negatively correlated with invertebrate grazer biomass in the field but positively related in the lab [53], suggesting that other factors may lead to conflicting responses depending on the experimental setting. Potential confounding factors in our field experiment may include slightly (but not significantly) higher guppy visitation rates in the downstream than the midstream reach or their interactions with some other unobserved consumer in the downstream reach (see Table S1). Both guppy phenotypes significantly reduced chlorophyll biomass relative to guppy-free treatments in mesocosms [28], suggesting that guppies may exploit suboptimal resources when preferred resources become limiting in artificial systems. However, non-predator-adapted guppies appear to be more flexible in their food selection than predator-adapted guppies in the presence of both wild and mesocosm-acclimated fish [27]. Although rare, the scavenging carnivorous freshwater eel, *Synbranchus marmoratus*, has been found previously in all three of our reach types [16], [17], [54] and thus, might also affect guppy diets. Combined, these studies suggest that the interspecific competition between guppies and killifish facilitate facultative feeding more in our midstream reach and the presence of predators may result in more rigid feeding preferences by guppies in our downstream reach.

Other potential contributors to the observed periphyton effects are biofilm grazing Loricariid catfish, *Hypostomus robinii* and *Ancistrus cirrhosus*, in the downstream reach. Although we never observed the catfish or their obvious feeding marks on substrates in our control frames, studies in similar systems show other grazing fish can significantly reduce periphyton biomass [55], [56] and even alter whole-stream carbon cycling [57]. Enhanced nutrient recycling rates in reaches with high consumer densities may also have alleviated local nutrient limitation in periphyton, which could act in opposition to direct consumer effects [13]. Bioturbation on tile periphyton is also possible in our upstream reach and to a lesser extent in the midstream reach where we observed killifish occasionally stopping on tiles and resuspending large quantities of loose organic material from tiles in control frames as they darted to another location. Bioturbation by killifish on tiles was probably not an issue in the downstream reach where their visitation was much lower than other reaches. Physical disturbance in exclosures was likely minimal relative to controls due to reduced access by large consumers. Based on visual observations, turbidity associated with consumer activities was localized mainly within control frames and dissipated quickly due to stream flow.

### Benthic Invertebrate Responses to Consumer Interactions

Consumers had the greatest positive impact (positive CI) on invertebrate abundance in the upstream reach (Fig. 2A). A 6-fold difference in ostracods in the presence of large consumers in the upstream reach explained most of the positive CI in benthic invertebrate abundance and suggests that killifish or crabs may facilitate ostracod survival and/or reproduction in control frames. Large predaceous dragonfly nymphs (Odonata) constitute about 1 to 2% of killifish diets [58], which may be enough to regulate these invertebrate predators in frames accessible to killifish. Because ostracods can constitute up to 50% of odonate diets [59], a reduction in odonate densities via killifish predation might explain the higher ostracod numbers we observed in control frames. More predation by odonates on ostracods could also help explain the greater periphyton biomass and accrual we observed in exclosures in the upstream reach. Crabs may also have played a role in increasing the number of smaller invertebrates in controls. Crabs in Neotropical mangrove forests can consume over 80% of litter production [60]. Because most of crab-processed leaf material is returned to the environment as feces or non-ingested fine detritus [61], crabs may enhance availability of fine organic matter for consumption by smaller invertebrate collectors, such as ostracods in control frames.

The greatest reduction in invertebrate abundances (negative CI), particularly among fine organic matter collectors, were observed in the downstream reach (Fig. 2A). Larger negative impacts on benthic invertebrates in the downstream relative to the midstream reaches (positive ΔCI in Fig. 2C) is consistent with the guppy diet differences mechanism described above and suggests that guppy phenotype may also be important in structuring benthic invertebrate composition in these streams. Two related studies, found that guppies from reaches with piscivores ate significantly more benthic invertebrates than guppies from reaches with killifish alone ([27], Table 1), but the degree of impact on benthic invertebrates is dependent on guppy density as well as phenotype in mesocosms [28]. Such intraspecific interactions are more likely to occur in midstream reaches where guppies typically have higher densities than in downstream reaches where their numbers are kept in check by piscivorous fishes [26]. Because guppy visitation to controls in midstream and downstream reaches were not significantly different in our study, greater consumer impact on invertebrates in the downstream reaches suggests guppies relatively free from interspecific competition with killifish were better able to reduce invertebrate abundances in the downstream reach.

Killifish may have also directly contributed to benthic invertebrate responses however, they generally occupy (and presumably feed in) flat complex river edges and riffles where guppies and predators are less dense in downstream reaches [59], whereas our experimental frames were placed in pools in the main stream channel. Killifish movement between reaches is positively related predator presence [62] and guppy competitors also appear to facilitate localized killifish exploratory behavior [63], but this was not likely to occur at large spatial scales between our reaches or with enough frequency to influence ecosystem responses over the duration of our experiment. The greater variety of large consumers and/or the necessity for our shorter experimental duration could also help explain the low total invertebrate abundance in downstream relative to the other reaches. Other omnivorous consumers in this reach included *Aequidens*, *Astyanax*, *Hemibrycon* and *Rhamdia* [16], although none of these fishes were observed in control frames in the downstream reach.

The change in direction of impact on benthic invertebrates between the upstream and downstream reaches suggests a switch in the fish consumer diets from terrestrial to aquatic prey or some competitive interaction among consumers for benthic resources. For example, terrestrial invertebrate input increases with increasing canopy cover and killifish diets tend to reflect prey availability in other Trinidadian streams [64], suggesting their impacts on benthic invertebrates are largely environment-dependent. However, fish treatments reduced benthic invertebrate biomass more in mesocosms when guppies and killifish are from sympatric assemblages relative to treatments when the killifish are naïve to guppies [65], suggesting competitive interactions between co-occurring fishes may also mediate impacts on benthic invertebrates in natural streams. Because killifish can apparently modify their diet from terrestrial to aquatic invertebrates, regardless of the mechanism (environmental and/or trophic), such a switch may also explain some of the resulting differences in other resources and process rates between reaches. For example, if terrestrial prey is scarce, a shift to benthic prey by killifish might result in accumulation of fine organic matter and/or algae that might have otherwise been consumed by benthic invertebrates.

### Multiple Roles of Consumers in Leaf Decay

Decomposition rates in controls were fastest in the upstream reach followed by the downstream and midstream reaches, respectively (Fig. 2B). Black stick leaf decay rates in these systems were relatively fast, but not abnormal for fresh leaves [35], [66]. There was also no longitudinal trend in leaf decomposition rates in a Portuguese stream system, but decomposition rates were also significantly faster when consumers had access to leaves in most upstream sites [67]. Other studies have found leaf decomposition covaries with many factors [68], particularly water temperature [69] and water velocity [70], but neither of these factors were significantly related to leaf decay in our study. The largest positive CI in our upstream reach underscores the role of consumers in tropical headwaters [3] and points to a major role of either crabs and/or killifish in facilitating leaf breakdown.

Crabs were directly observed shredding plant debris in our reaches and had the highest average visitation rates in reaches corresponding with the fastest leaf decomposition rates. Although crab visitation across all reaches was not significantly related to CI for leaf decomposition (Fig. 3, hollow symbols), crabs do obtain much of their carbon from leaves [71] and facilitate leaf breakdown by physically shredding leaves [72] in other studies. The leaf-shredding caddisfly, genus *Phylloicus*, was also most abundant in benthic samples from both exclosure and control frames in the upstream reach, but was only occasionally observed in leaf pack samples. Killifish visitation to control frames was positively related to the CI for leaf decomposition rate (Fig. 3, solid symbols), and thus may contribute to leaf breakdown directly through associated bioturbation of these labile fresh leaves. Variation in consumer distributions can also indirectly enhance local biogeochemical processes [73]. For example, killifish using leaves for refuges may also facilitate decay indirectly by elevating nutrient availability via excretion to leaf-associated heterotrophic microbes [74]. Higher ambient nutrient concentrations in the downstream reach (Table S2) could also enhance leaf-associated microbes in both treatment types, which would result in faster decomposition rates downstream than other reaches. This was the case for exclosure leaf decomposition downstream (Table 3), but nutrient concentrations alone do not fully explain why the fasted decomposition rates occurred in controls in the upstream reach.

The absence of a consumer effect on leaf decay in the midstream compared to a significant effect in the upstream reach is striking, given the main assemblage difference is the presence of guppies in the midstream reach (Fig. 2D). We observed guppies pecking on leaf packs in control frames in midstream and downstream reaches, but this was likely in pursuit of leaf-associated invertebrates [28] rather than direct leaf matter consumption by guppies, which could result in increased leaf decay rate. Competitive and predatory interactions between guppies and killifish could also possibly contribute to differences in leaf decomposition. For example, killifish densities are reduced by as much as 75% and growth rates are reduced in reaches where they co-occur with guppies [16], [75]. In addition, adult guppies appear to prey on young killifish [24] and killifish prey on smaller guppies [76], but the net effect of these trophic interactions generally results in a negative impact on killifish densities. Reduced killifish densities in the presence of guppies would thus reduce the direct and indirect effects described above and ultimately reduce leaf decay rates as we observed in the midstream reach.

In conclusion, we found that the roles of large consumers in this Neotropical stream system appears to correspond with variation in their local distribution, interactions with other consumers in the assemblage (Table S1) and potentially local adaptation to those interactions. Because life history evolution can occur rapidly [77], factors like local adaptation in key species must also be incorporated into our evaluation of consumer effects in natural ecosystems. Subjected to changes in predator regime, populations have the potential to develop not only changes in diet [27], but also degree of impact on the surrounding environment and associated resources [28]. There is considerable niche differentiation between guppies and killifish particularly in downstream reaches where piscivore presence strongly mediates killifish movement and habitat selection [63]. Competition between guppies and killifish may also have broad implications for ecosystem structure and processes in Trinidadian streams [66]. For example, we found that in reaches where they do not occur with guppies, killifish are linked with reduced periphyton accrual and biomass and faster leaf decomposition rates. When they co-occur with guppies, killifish have much lower densities [16] but faster growth rates [78], which likely led to large between-reach differences in impact on leaf decomposition observed in our study (Fig. 2D). The degree of top-down effect exerted by killifish in our streams therefore appears to be largely mediated by the presence of guppies. The ecological effects of different species with similar trophic niches can be particularly difficult to predict when they co-occur in the same location [15]. By running manipulative experiments in reaches characterized by overlapping assemblages, we have helped reveal some of the potential effects of the common large consumers on the structure and processes of this Neotropical stream ecosystem.

## Supporting Information

Methods S1 **Statistical methods for split-plot design and analytical framework for structural and process responses.**
(DOC)

Table S1 **Consumer species, feeding group and relative abundance of eleven fishes and one crab occurring in study reaches.**
(DOC)

Table S2 **Site characteristics at the three study reaches.**
(DOC)

Table S3 **Benthic invertebrate tallies for control and electrified treatments in three reaches.**
(DOC)
