# Supplementary material for: Effects of Consumer Interactions on Benthic Resources and Ecosystem Processes in a Neotropical Stream
Source: PLoS One. 2012 Sep 28;7(9):e45230. doi: 10.1371/journal.pone.0045230 (PMC3461008; doi:10.1371/journal.pone.0045230)
Supplement: Methods S1 — Statistical methods for split-plot design and analytical framework for structural and process responses. (DOC) [file pone.0045230.s001.doc]

Methods S1. For structural response variables (week 3 periphyton chlorophyll *a* and AFDM, and final invertebrate abundances and biomasses), we used reach (upstream, midstream, downstream) as our whole plot and exclosure type as our split plot fixed effects. We modeled pool number nested within reach (whole plot) as a random effect to identify the pool as our independent subject for the whole plot effects. Because an initial analysis revealed strong heterogeneity of variances among treatments, we modeled the separate residual errors for either the reach or exclosure effects to obtain a better overall model fit to the data as evaluated by likelihood-ratio tests. All dependent variables were natural log-transformed prior to analysis to facilitate consumer contrasts (see below) and to reduce errors that were correlated with the mean predicted values.

For response variables that we measured through time (leaf decomposition rates and periphyton accrual rates) this analytical framework was modified so that we could calculate and test differences between slopes as well as categorical effects between treatments. For periphyton accrual rates, we included day of the experiment as a covariate and interactions between day and each of the other fixed effects and fixed effects interactions. We also added the interaction between day and pool nested within reach as a random effect and an unstructured covariance matrix to calculate the proper error term for whole-plot slope effect. We centered the day covariate on day 20 so that all omnibus *F*-tests of fixed effects and contrasts not including the covariate were evaluated at day 20 of the experiment. This represents tests of periphyton biomass after 20 days. We also constructed linear contrasts to test the slope differences (periphyton accrual rates) between exclosure types within reaches and between reaches. These contrasts are identical to the contrasts for the response variables measured once, except that they now include the interaction between day and exclosure type as well as the interaction between day, exclosure type, and reach. Measurements of chlorophyll *a* were natural log-transformed prior to analysis, which as for the single measurement variables results in contrasts that represent consumer indices for the fixed effects (periphyton biomass), but not for the slope contrasts (periphyton accrual rates). Therefore the difference contrast tests are the differences between the untransformed slope values. To facilitate comparison with other variables, we calculated the consumer index for each reach and the differences between reaches using the predicted slope value for each exclosure replicate for all three reaches using the formula as -ln(electrified/non-electrified).

We used a similar approach to analyzing leaf decomposition rates. We used a slope-only model (no fixed effects and curve forced through the origin) with natural log-transformed percent remaining dry mass as a dependent variable and day and the interactions between day and reach, treatment, and reach by treatment interaction as covariate effects. The interaction between day and pool nested within reach was included as a random effect. The resulting slope values represent the decomposition rates (k), which is the factional leaf mass loss per day [S1]. Contrasts and calculation of consumer indices were as for periphyton accrual rates.

Supplementary References

S1. Benfield EF (2006) Decomposition of leaf material. In: Hauer FR, Lamberti GA, editors. Methods in Stream Ecology, Academic Press, Burlington, Massachusetts, USA. pp. 711–720.
