# Supplementary material for: Effects of Consumer Interactions on Benthic Resources and Ecosystem Processes in a Neotropical Stream
Source: PLoS One. 2012 Sep 28;7(9):e45230. doi: 10.1371/journal.pone.0045230 (PMC3461008; doi:10.1371/journal.pone.0045230)
Supplement: Table S1 — Consumer species, feeding group and relative abundance of eleven fishes and one crab occurring in study reaches. (DOC) [file pone.0045230.s002.doc]

Table S1.

|  |  | Relative Abundance | | |
| --- | --- | --- | --- | --- |
| Species (common name) | Feeding Group | UP | MID | DOWN |
| *Rivulus hartii* (killifish) | carnivorous-insects/fish | C | I | R |
| *Rhamdia quelen* (catfish) | carnivorous-insects |  | R | I |
| *Poecilia reticulata* (guppy) | omnivorous-periphyton/insects |  | C | C |
| *Hoplias malabaricus* (wolf fish) | carnivorous-fish |  |  | I |
| *Hypostomus robinii* (momma teta) | omnivorous-periphyton |  |  | I |
| *Ancistrus cirrhosus* (jumbie teta) | omnivorous-periphyton |  |  | I |
| *Aequidens pulcher* (blue cocsarob) | carnivorous-insects/fish |  |  | I |
| *Cichlasoma taenia* (brown coscarob) | carnivorous-insects/fish |  |  | I |
| *Astyanax bimaculatus* (red-fin sardine) | omnivorous-plants/seeds/insects/algae |  |  | C |
| *Hemibrycon taeniurus* (mountain stream sardine) | omnivorous-insects/fish/seeds |  |  | C |
| *Synbranchus marmoratus* (eel) | carnivorous-carrion/slow prey | R | R | R |
| *Eudaniela garmani* (crab) | omnivorous-detritus | C | I | C |

Fish species presence from [16]

Fish feeding group from [17,27, and Taphorn DC (1992) The Characiform fishes of the Apure River drainage , Venezuela.  Biollania Edition number 4, UNELLEZ, Guanare, Venezuela.]

Relative abundances (C=common, I=infrequent, R=rare) based on visitation observations within control frames (Table 3) or qualitative visual surveys in each reach during experiment sampling days. C: observed in two or more control frames during visitation observations on more than one date; I: observed in more than one frame during visitation observations on only one date or in nearby habitats during qualitative surveys; R: observed in only one control frame during visitation on only one date or not observed at all during qualitative surveys, but previously found in other quantitative studies in nearby locations.

Stream reach abbreviations: UP=upstream, MID=midstream, DOWN=downstream.
