# Supplementary material for: Effects of Consumer Interactions on Benthic Resources and Ecosystem Processes in a Neotropical Stream
Source: PLoS One. 2012 Sep 28;7(9):e45230. doi: 10.1371/journal.pone.0045230 (PMC3461008; doi:10.1371/journal.pone.0045230)
Supplement: Table S2 — Site characteristics at the three study reaches. (DOC) [file pone.0045230.s003.doc]

Table S2.

|  | Reach | | |
| --- | --- | --- | --- |
| Variable | Upstream | Midstream | Downstream |
| Dates of Experiment | 8-Feb to 7-Mar | 7-Feb to 6-Mar | 5-Apr to 26-Apr |
| Stream Order | 2 | 2 | 4 |
| Elevation (m) | 210 | 200 | 140 |
| Reach Length (m) | 200 | 200 | 150 |
| Canopy cover (%) | 76.1(3.8) | 85.5(1.4) | 77.5(2.1) |
| Water Temperature (ºC) | 22.4(0.05) | 22.4(0.02) | 25.5(0.04) |
| Dissolved O_2_ (mg O_2_·L^-1^) | 7.7(0.04) | 7.6(0.02) | 8.3(0.11) |
| Percent Dissolved O_2_ | 88.8(1.4) | 87.6(1.2) | 95.6(2.2) |
| Water Velocity (m·s^-1^) | 0.04(0.006) | 0.05(0.007) | 0.014(0.008) |
| Water Depth (cm) | 21.2(2.5) | 23.1(2.5) | 22.3*(3.0) |
| NH_4_^+^ (μg N·L^-1^) | 0.3(0.08) | 0.3(0.08) | 3.1(0.15) |
| SRP (μg P·L^-1^) | 16.5(0.20) | 16.5(0.20) | 23.4(0.59) |

All values are means (±1standard error)

Canopy cover was measured with a spherical densiometer.

Water temperature and dissolved oxygen was measured with a YSI 85 meter.

Water velocity was measured with a Marsh-McBirney current meter.

Nutrient data is based on samples taken in July 2008. SRP = soluble reactive phosphorus

*Estimated from photos taken during sampling.
