# Supplementary material for: Effects of Consumer Interactions on Benthic Resources and Ecosystem Processes in a Neotropical Stream
Source: PLoS One. 2012 Sep 28;7(9):e45230. doi: 10.1371/journal.pone.0045230 (PMC3461008; doi:10.1371/journal.pone.0045230)
Supplement: Table S3 — Benthic invertebrate tallies for control and electrified treatments in three reaches. (DOC) [file pone.0045230.s004.doc]

Table S3

|  |  |  | Upstream | | Midstream | | Downstream | |
| --- | --- | --- | --- | --- | --- | --- | --- | --- |
| Class | Order | Family | C | E | C | E | C | E |
| Annelida | Oligochaeta | unknown | 0 | 0 | 1 | 1 | 0 | 0 |
| Arachnida | Hydracarina | unknown | 19 | 33 | 27 | 28 | 12 | 3 |
| Crustacea | Amphipoda | unknown | 1 | 0 | 0 | 0 | 0 | 0 |
| Crustacea | Copepoda | Harpactocoida | 0 | 0 | 0 | 0 | 4 | 1 |
| Crustacea | Copepoda | unknown | 2 | 0 | 0 | 0 | 0 | 0 |
| Crustacea | Ostracoda | unknown | 475 | 77 | 417 | 239 | 41 | 36 |
| Insecta | Coleoptera | Elmidae | 16 | 16 | 0 | 0 | 36 | 46 |
| Insecta | Coleoptera | Psephenidae | 4 | 1 | 2 | 4 | 31 | 15 |
| Insecta | Coleoptera | unknown | 0 | 4 | 0 | 0 | 0 | 0 |
| Insecta | Diptera | Blephariceridae | 0 | 0 | 1 | 1 | 0 | 0 |
| Insecta | Diptera | Ceratopogonidae | 52 | 29 | 35 | 28 | 22 | 54 |
| Insecta | Diptera | Chaoboridae | 0 | 0 | 0 | 0 | 0 | 1 |
| Insecta | Diptera | Chironomidae | 692 | 570 | 782 | 794 | 58 | 76 |
| Insecta | Diptera | Culicidae | 4 | 1 | 0 | 0 | 0 | 0 |
| Insecta | Diptera | Muscidae | 2 | 1 | 0 | 0 | 0 | 0 |
| Insecta | Diptera | Psychodidae | 5 | 8 | 2 | 1 | 0 | 0 |
| Insecta | Diptera | Simuliidae | 1 | 0 | 1 | 1 | 0 | 0 |
| Insecta | Diptera | Stratyomidae | 0 | 1 | 0 | 0 | 0 | 0 |
| Insecta | Diptera | Tipulidae | 1 | 0 | 0 | 0 | 0 | 0 |
| Insecta | Diptera | unknown | 1 | 3 | 0 | 0 | 0 | 0 |
| Insecta | Ephemeroptera | Baetidae | 9 | 5 | 1 | 2 | 4 | 4 |
| Insecta | Ephemeroptera | Euthyplocidae | 1 | 1 | 0 | 1 | 0 | 0 |
| Insecta | Ephemeroptera | Leptohyphidae | 34 | 7 | 13 | 5 | 156 | 111 |
| Insecta | Ephemeroptera | Leptophlebiidae | 2 | 2 | 0 | 0 | 2 | 4 |
| Insecta | Ephemeroptera | unknown | 2 | 0 | 0 | 0 | 7 | 16 |
| Insecta | Hymenoptera | Formicidae | 0 | 3 | 0 | 0 | 0 | 0 |
| Insecta | Hymenoptera | unknown | 0 | 1 | 0 | 0 | 0 | 0 |
| Insecta | Megaloptera | Corydalidae | 0 | 2 | 0 | 0 | 0 | 0 |
| Insecta | Neuroptera | unknown | 0 | 1 | 0 | 0 | 0 | 0 |
| Insecta | Odonata | Coenagrionidae | 1 | 2 | 1 | 0 | 1 | 2 |
| Insecta | Odonata | Libellulidae | 4 | 3 | 1 | 0 | 1 | 5 |
| Insecta | Plecoptera | Perlidae | 0 | 0 | 1 | 2 | 0 | 0 |
| Insecta | Psocoptera | unknown | 1 | 0 | 0 | 0 | 0 | 0 |
| Insecta | Trichoptera | Calamoceratidae | 5 | 4 | 0 | 1 | 0 | 0 |
| Insecta | Trichoptera | Glossosomatidae | 0 | 0 | 1 | 0 | 0 | 0 |
| Insecta | Trichoptera | Hydropsychidae | 0 | 1 | 33 | 26 | 0 | 5 |
| Insecta | Trichoptera | Leptoceridae | 3 | 4 | 3 | 3 | 1 | 0 |
| Insecta | Trichoptera | Philopotamidae | 0 | 1 | 1 | 0 | 0 | 0 |
| Insecta | Trichoptera | Polycentropidae | 0 | 0 | 0 | 0 | 1 | 0 |
| Insecta | Trichoptera | unknown1 | 0 | 0 | 0 | 0 | 0 | 10 |
| Insecta | Trichoptera | unknown2 | 0 | 0 | 0 | 0 | 0 | 1 |
| Insecta | unknown | unknown | 0 | 1 | 0 | 0 | 0 | 0 |
| Nematomorpha | unknown | unknown | 0 | 0 | 0 | 1 | 0 | 0 |
| Platyheminthes | Hirudinea | unknown | 0 | 0 | 0 | 1 | 0 | 0 |
| Rotifera | unknown | unknown | 0 | 0 | 0 | 0 | 0 | 1 |

Tallies from benthic invertebrate samples collected at the end of the experiment period in each reach and each treatment (C, control; E, exclosure). Units are total number of individuals found in all pipe samples from 5 treatment replicates (total area = 0.0458 m^2^). One individual each from families of Trichoptera (Leptoceridae, Polycentropidae, Glossosomatidae, Philopotamidae), Odonata (Coenagrionidae, Libellulidae) and Diptera (Simuliidae, Tipulidae) were observed in one control frame from one reach, but were not observed in any exclosures in that same reach. These exceptions constitute 0.3% of the 3035 total individuals in control samples and 0.4% of the 2312 total individuals in exclosure samples. Leptocerids, Coenagrionids and Libellulids were also observed in both control and electrified treatment samples in the other two reaches. Polycentropids, Glossosomatids and Tipulids were not observed in either treatment in the other two reaches. Philopotamids were also observed in 1 exclosure treatment in one other reach. Simuliids were observed in both treatment types in one other reach.
